# Factors Associated with Clinical and Topographical Features of Laryngeal Tuberculosis

**DOI:** 10.1371/journal.pone.0153450

**Published:** 2016-04-14

**Authors:** João Gustavo Corrêa Reis, Clarissa Souza Mota Reis, Daniel César Silva da Costa, Márcia Mendonça Lucena, Armando de Oliveira Schubach, Raquel de Vasconcellos Carvalhaes Oliveira, Valéria Cavalcanti Rolla, Fátima Conceição-Silva, Cláudia Maria Valete-Rosalino

**Affiliations:** 1 Evandro Chagas National Institute of Infectious Diseases (INI), Oswaldo Cruz Foundation (FIOCRUZ), Rio de Janeiro, RJ, Brazil; 2 Laboratory of Immunoparasitology, Oswaldo Cruz Institute (IOC), FIOCRUZ, Rio de Janeiro, RJ, Brazil; 3 Department of Bronchoesophagolaryngology and Head and Neck Surgery, Bonsucesso Federal Hospital, Rio de Janeiro, RJ, Brazil; 4 Department of Otorhinolaryngology and Ophthalmology, Faculty of Medicine, Federal University of Rio de Janeiro, Rio de Janeiro, Brazil; Food and Drug Administration, UNITED STATES

## Abstract

**Introduction:**

Laryngeal tuberculosis (LTB) is the most frequent granulomatous disease of the larynx and represents less than 2% of extrapulmonary TB cases. There are no pathognomonic clinical and endoscopic features of this disease and studies on LTB that can assist in its diagnostic characterization are lacking.

**Objective:**

To identify factors associated with clinical and topographical features of LTB.

**Method:**

a retrospective cross-sectional study was conducted from the medical records of 36 patients with confirmed LTB diagnosis.

**Results:**

Dysphonia and cough were the main symptoms presented by patients and the true vocal folds the most frequently affected site. The average of the duration of the disease evolution was significantly higher in patients with dysphonia than in patients without this symptom. We observed association between dysphonia and true vocal fold lesions and between odynophagia and lesions in the epiglottis, arytenoids and aryepiglottic folds. Odynophagia was more frequent in individuals with lesions in four or more laryngeal sites. Weight loss equal or above 10% of the body weight was more frequent in patients with odynophagia as first symptom and in patients with ulcerated lesion. Dyspnea on exertion was more frequent in individuals with more extensive laryngeal lesions. The percentage of smokers with lesions in four or more laryngeal sites was greater than that found in non-smokers. Laryngeal tissue fragment bacilloscopy and culture examinations were less positive than sputum ones.

**Conclusions:**

Smoking appears to be associated with the development of more extensive LTB lesions, and LTB with dyspnea on exertion and odynophagia with consequent impairment of nutritional status. We emphasize the need for histopathologic confirmation, once positive sputum bacteriological examinations seem not to necessarily reflect laryngeal involvement.

## Introduction

Tuberculosis (TB) is a contagious infectious disease of chronic evolution caused by *Mycobacterium tuberculosis*. Despite progress in relation to the goals of disease control, TB is still a global public health problem [1,2]. Nine million new cases of this disease were registered in the world in 2013 by the World Health Organization (WHO) and of these, 83,310 cases were reported in Brazil, which is one of the 22 countries prioritized by WHO, because they concentrate 80% of TB global burden. Additionally, following the emergence of the Human Immunodeficiency Virus (HIV), it has become the second leading cause of death by a single infectious agent. In 2013, of 1.5 million people who died of TB, 360,000 were HIV-positive [2].

TB affects mainly the lungs but can occur in any organ. Laryngeal tuberculosis (LTB) represents less than 2% of extrapulmonary TB cases [3,4] and is the most frequent granulomatous disease of this organ [5–7]. Literature data indicates that LTB incidence rate among patients diagnosed with pulmonary TB varies between 0.08 and 5.1% [8–10]. However, the exact incidence of LTB in patients with pulmonary TB is difficult to be determined because systematic otorhinolaryngologic evaluation of these patients is not usually conducted and it is likely that this disease is more frequent than diagnosed [8,11,12]. The hypothesis of LTB diagnosis is rarely considered by otorhinolaryngologists, which may delay its diagnosis and consequently increase the incidence of complications [10,13].

TB can affect any laryngeal anatomic site, with variable clinical and endoscopic features [12,14]. Skin tests and sputum analyses are considered auxiliary methods in LTB diagnosis [15,16]. On the other hand, histopathologic and microbiological analyses of laryngeal lesion fragments obtained by biopsy are essential for the diagnosis [7,12, 16] and differentiation from other chronic diseases such as neoplasia [3,8,16], other granulomatous infectious diseases such as leishmaniasis and paracoccidioidomycosis [14], and non-infectious granulomatous diseases such as Wegener’s granulomatosis and amyloidosis [5,17].

To date, there is a lack of studies on LTB that can assist in the diagnostic characterization of the disease. The objective of this study is to identify factors associated with clinical and topographical features of LTB.

## Material and Methods

This study was approved by the Ethics in Research Committee of the National Institute of Infectious Diseases (INI)–Oswaldo Cruz Foundation (FIOCRUZ) under protocol number 12243513.2.0000.5262 and a consent form was signed by all the patients. A retrospective cross-sectional study was conducted from the medical reports of LTB patients diagnosed by the presence of videolaryngoscopic signs of chronic laryngitis associated with mycobacterium identification by at least one of the following methods: sputum bacilloscopy or culture and/or analysis of tissue fragments obtained by laryngeal biopsy through direct examination, culture or histopathologic examination with Wade staining technique.

All patients were treated at the outpatient clinics for tuberculosis and otorhinolaryngology of INI- FIOCRUZ from 2004 to 2014 and monitored by a previously defined protocol. As exclusion criterion we used presence of immunosuppression or lack of information in the medical records.

The sociodemographic profile of the patients was evaluated using the following variables: age, gender, economic status, education level, comorbidities, smoking and drinking habits. Patients who had the habit of smoking daily regardless of the amount were considered smokers and those who consumed any alcoholic beverage and gave a positive response to at least two questions in the CAGE questionnaire were considered drinkers [18].

Clinical variables included signals, symptoms and endoscopic features of lesions identified by videolaryngoscopy using a 70 degree rigid videolaryngoscope (Karl Storz, Germany), video camera (Toshiba, Japan), video recorder (LG, USA) and video monitor (Sony, USA).

All LTB patients who presented signals of active infectious focus in the lungs through clinical examination and chest X-ray were considered as having pulmonary TB.

**Fig 1:**
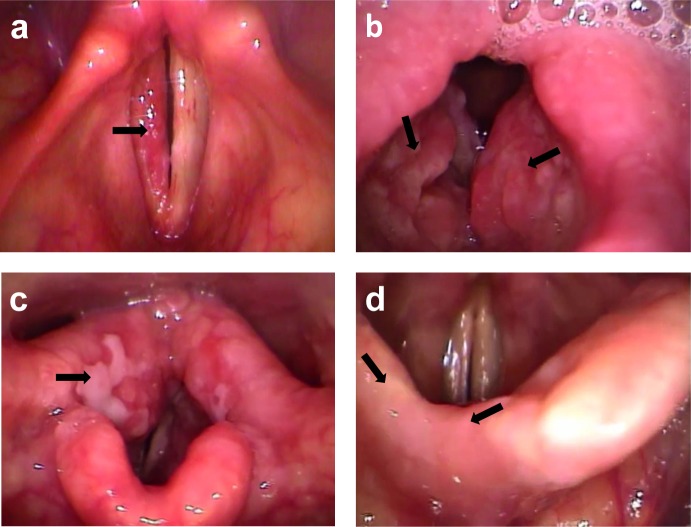
**Pictures of images obtained by Videolaryngoscopy**: a- Hyperemic and flat lesion, with smooth surface of right true vocal fold—example of nonspecific inflammatory lesion. b- Hyperemic lesion, with exophytic appearance with rough surface located in the false vocal folds–example of granulomatous lesion. c- Example of ulcerated lesion with fibrin located in aryepiglottic fold and right arytenoid region.d- Example of erosive lesion of the right half of the epiglottis. Source: Files of the Department of Otorhinolaryngology of the Evandro Chagas National Institute of Infectious Diseases (INI)-FIOCRUZ.

For a better understanding and topographical analysis of LTB involvement we divided the larynx in the following anatomic sites: epiglottis, aryepiglottic fold, arytenoid region, interarytenoid region, false vocal folds, true vocal folds and subglottis. To assess the extent of the lesions we used two variables classified as follows: unilateral or bilateral involvement; number of affected laryngeal sites (up to three affected sites or four or more affected sites). Since there is no standardization of the endoscopic description of LTB lesions in the literature [16], we defined four categories of videolaryngoscopic appearances: nonspecific inflammatory lesion (hyperemic lesion with flat or exophytic appearance with smooth surface) (Fig 1A); granulomatous lesion (hyperemic lesion with exophytic appearance with rough surface) (Fig 1B); ulcerated lesion (Fig 1C) and erosive lesion (Fig 1D).

Complementary tests included: chest X-ray, skin test with “Purified Protein Derivative” (PPD), considering the result as positive when induration was equal or above 5mm; analysis of sputum and tissue fragments (obtained by laryngeal biopsies) for acid-alcohol resistant bacillus (BAAR) detection through the Ziehl-Neelsen technique with assessment of bacterial load (0, +, ++, +++) [19] and culture in Lowenstein-Jensen/Ogawa-Kudoh medium and by the Mycobacteria Growth Indicator Tube (MGIT) system, determining bacterial growth load (0,+, ++, +++) [19]; and histopathologic examination of the same tissue specimens through 5 *μ*m sections stained with hematoxylin-eosin and Wade stain, examined under an optical microscope (Zeiss, Jena, Germany). Direct examination and culture bacterial loads, from both, sputum and laryngeal specimens were categorized into two groups; one with load up to two plus signs and the other with loads of three plus signs.

The Statistical Package for Social Science for Windows (SPSS) program version 16.0 (SPSS Inc., Chicago, IL, USA) was used for data analysis. The simple frequencies of the categorical variables were described as well as the summary measures (mean ± standard deviation (SD), median, interquartile range (IQR, minimum and maximum) of the continuous variables. The association between categorical variables was verified by Fisher’s exact test. The Shapiro-Wilk normality test indicated escape from normality of the variable time of laryngeal symptoms up to LTB diagnosis and, for the variable age, indicated non-rejection of normality. The Mann-Whitney test was used to compare the median times of laryngeal symptoms, whereas the T test was used to compare mean age by gender. P-values < 0.05 indicated significant differences.

## Results

Of 41 individuals initially identified with LTB, three (7.3%) patients were excluded because of co-infection with HIV or concomitant immunosuppressive therapy and two (4.9%) because of lack of information in the medical records. Thirty six patients with LTB were included. The age of the individuals at diagnosis ranged from 22 to 82 years, with mean of 47.08 ± 14.75 years. Twenty-eight (77.8%) patients were male (gender ratio 3.5:1) and there was no age difference between men and women (p = 0.460). Other clinical and bacteriological features of the patients are described in Table 1.

**Table 1:** Frequencies of categorical variables related to the epidemiological and clinical characteristics of 36 patients with laryngeal tuberculosis diagnosed at the Evandro Chagas National Institute of Infectious Diseases—FIOCRUZ, from 2004 to 2014.

Variable		n	%
Gender	male	28	77.8
	female	8	22.2
Monthly income	up to US$ 590	30	83.3
	more than US$ 590	6	16.7
Education level	primary school completed	24	66.7
	high school or more	12	33.3
Past medical history	*diabetes mellitus*	4	11.1
	previous pulmonary tuberculosis	6	16.7
	contact with pulmonary tuberculosis	9	25
Habits	only smoking	8	22.2
	only alcohol drinking	5	13.9
	concomitant smoking and alcohol use	8	22.2
	neither smoking nor alcohol use	15	41.7
Symptoms	dysphonia	32	88.9
	cough	32	88.9
	odynophagia	27	75
	dyspnea on exertion	19	52.8
	dysphagia	11	30.6
	dyspnea at rest	5	13.9
	reflex otalgia	2	5.6
First symptom	dysphonia	26	72.2
	odynophagia	13	36.1
	cough	12	33.3
	dyspnea	2	5.6
	dysphagia	1	2.8
Affected laryngeal sites	epiglottis	21	58.3
	aryepiglottic fold	22	61.1
	arytenoid region	18	50
	interarytenoid region	12	33.3
	false vocal fold	24	66.7
	true vocal fold	32	88.9
Number of affected sites	1 up to 3	14	38.9
	4 or more	22	61.1
Number of affected sides	unilateral	8	22.2
	bilateral	28	77.8
Endoscopic appearance	granulomatous lesion	24	66.7
	nonspecific inflammatory lesion	19	52.8
	ulcerated lesion	14	38.9
	erosive lesion	8	22.2

n—number of patients

The interval between the onset of laryngeal symptoms and diagnosis was between one and 36 months, with median of five and a half months (IQR = 3–12). All patients were symptomatic with one to six symptoms (median = 4). Weight loss equal or above 10% of body weight was observed in 19 (52.8%) patients.

The simultaneous involvement of more than one larynx anatomic structure was identified in 31 (86.1%) patients and the extent of the lesion to the pharynx in seven (19.4%). In the five (13.9%) cases restricted to a single laryngeal site, the inflammatory process was located in the vocal folds. Erosive lesions, when present, were identified only in the epiglottis. Regarding vocal fold motility, two (5.6%) patients had unilateral paresis, two (5.6%) unilateral paralysis and one (2.8%) bilateral paralysis and laryngeal stenosis. The later underwent tracheostomy.

Results of complementary examinations are shown in Table 2. In 35 patients (97.2%) with radiological abnormalities compatible with pulmonary TB, there were changes on auscultation that corroborated this diagnosis. Involvement of four or more laryngeal sites was observed in 50% patients with positive PPD and in all patients with negative PPD. There was no association between the duration of LTB evolution, location, endoscopic appearance, laryngeal lesion extent degree and PPD results (p>0.05). No associations were found between bacterial loads (in BAAR and culture) of sputum or laryngeal tissue specimens with smoking habits, alcohol consumption, symptoms related to TB, extension of laryngeal involvement, endoscopic appearance of the lesion and PPD (p>0.05).

**Table 2:** Frequency of complementary examination results in 36 patients with laryngeal tuberculosis treated at the Evandro Chagas National Institute of Infectious Diseases- FIOCRUZ, from 2004 to 2014.

Complementary examination	Results	n	%
Chest X-ray suggesting pulmonary tuberculosis (N = 36)	yes	35	97.2
	no	1	2.8
Skin test with Purified Protein Derivative (PPD)	positive	24	85,7
	negative	4	14,3
Determination of BAAR in sputum by Ziehl-Neelsen	positive	29	80.6
technique (N = 36)	negative	7	19.4
BAAR load in sputum (N = 29) *	+	13	36.1
	++	9	25.0
	+++	7	19.4
Sputum culture (N = 35)	positive	33	94.3
	negative	2	5.7
Load of bacterial growth in sputum culture (N = 31)**	+	9	25.0
	++	9	25.0
	+++	15	41.7
Determination of BAAR in laryngeal biopsy tissues	positive	2	14.3
(by Ziehl-Neelsen technique) (N = 14)	negative	12	85.7
Culture of laryngeal biopsy tissues (N = 12)	positive	6	50.0
	negative	6	50.0
Histopathologic findings—inflammatory process (N = 19)	granulomatous	17	89.5
	nonspecific	2	10.5
Determination of BAAR in laryngeal biopsy tissues	positive	8	50.0
(by Wade stain) (N = 16)	negative	8	50.0

n—number of patients

N—valid numbers

BAAR—Acid-alcohol resistant bacillus

*—+: 10 to 99 BAAR in 100 fields; ++: average of 1 to 10 BAAR per field in the first 50 fields observed; +++: average of more than 10 BAAR per field in the first 20 fields observed

**—+: 20 to 100 colonies; ++: more than 100 separate colonies; +++: confluent colonies

The single LTB patient without detectable pulmonary involvement presented a lesion with the same features of most of the other LTB patients (bilateral granulomatous lesion at the true vocal folds, without ulcer or paralysis). Dysphonia was the first and main symptom. This patient had negative bacilloscopy as well as negative culture of sputum and laryngeal specimen. The diagnosis was confirmed only by the presence of a few bacteria identified in laryngeal tissue by Wade stain. In addition, the histopathology of the laryngeal specimen showed a chronic granulomatous inflammatory process.

No associations were found between the presence of dysphagia and cough with endoscopic appearance and lesion location (p>0.05). All patients with lesions in four or more sites had cough. In addition, 27 (84.4%) patients with cough and only one (25%) of the patients without cough presented bilateral involvement (p = 0.028).

The median time for disease evolution at LTB diagnosis of patients with dysphonia (6 months) was significantly higher than that of patients without this symptom (2.5 months) (p = 0.038). On the other hand, no significant difference was observed of the median time of disease progression in relation to presence or absence of other symptoms (p>0.05). In addition, this median time was not significantly different between the groups of individuals with uni or bilateral lesions, in relation to the presence or absence of each endoscopic appearance and between the patients grouped by the number of involved sites (p>0.05).

The epiglottis was the only laryngeal site associated with the endoscopic appearance of the lesion. The frequency of the ulcerated lesion in patients with lesion in the epiglottis (57.1%) was higher than that found in patients without lesions in this anatomic site (13.3%) (p = 0.008). Additionally, the endoscopic appearance of the lesions was not associated with laryngeal symptoms (p>0.05).

The distribution of dysphonia and odynophagia symptoms according to the anatomic sites (laryngeal topographical features) is described in Tables 3 and 4 respectively.

**Table 3:** Comparison of the presence or not of dysphonia with topographical features of the lesions of 36 patients with laryngeal tuberculosis. Evandro Chagas National Institute of Infectious Diseases—FIOCRUZ, 2004 to 2014.

	Presence of dysphonia				
	Yes		No		
	(N = 32)		(N = 4)		
Laryngeal topographical features	n	%	n	%	p-valueª
Lesions in the epiglottis	17	53.1	4	100	*
Lesions in the aryepiglottic fold	19	59.4	3	75.0	1.000
Lesions in the arytenoid region	16	50.0	2	50.0	1.000
Lesions in the interarytenoid region	9	28.1	3	75.0	0.098
Lesions in the false vocal fold	24	75.0	0	0.0	**
Lesions in the true vocal fold	31	96.9	1	25.0	**0.002**
Lesions in the subglottic region	4	12.5	0	0.0	**
Lesions in four or more sites	20	62.5	2	50.0	0.634
Bilateral lesions	25	78.1	3	75.0	1

N—total number, n—valid number, **bold—significant p value**

ª - p-value calculated by the Fisher`s Exact Test

*—association analysis was not possible due to the presence of lesion in the epiglottis in all patients without dysphonia

**—association analysis was not possible due to absence of lesion in the false vocal fold and subglottic region in patients without dysphonia

**Table 4:** Comparison of the presence or not of odynophagia with topographical features of the lesions of 36 patients with laryngeal tuberculosis. Evandro Chagas National Institute of Infectious Diseases—FIOCRUZ, 2004 to 2014.

	Presence of odynophagia				
	Yes		No		
	(N = 27)		(N = 9)		
Laryngeal topographical features	n	%	n	%	p-valorª
Lesions in the epiglottis	19	70.4	2	22.2	**0.019**
Lesions in the aryepiglottic fold	21	77.8	1	11.1	**0.001**
Lesions in the arytenoid region	17	63.0	1	11.1	**0.018**
Lesions in the interarytenoid region	11	40.7	1	11.1	0.219
Lesions in the false vocal fold	20	74.1	4	44.4	0.126
Lesions in the true vocal fold	23	85.2	9	100	*
Lesions in the subglottic region	3	11.1	1	11.1	1.000
Lesion extended to the pharynx	7	25.9	0	0.0	**
Lesions in four or more sites	21	77.8	1	11.1	**0.001**
Bilateral lesions	24	88.9	4	44.4	**0.013**

N—total number, n—valid number, **bold—significant p value**

ª - p-value calculated by the Fisher`s Exact Test

*—association analysis was not possible due to the presence of lesions in vocal folds in all patients without odynophagia

**—association analysis was not possible due to the presence of odynophagia in all patients with lesion extended to the pharynx

Weight loss equal or above 10% of body weight was observed in 76.9% patients with odynophagia as first symptom and in only 39.1% patients without this initial symptom (p = 0.029). The frequency of ulcerated lesions in patients with weight loss equal or above 10% (57.9%) was higher to that found in patients without this sign (17.6%) (p = 0.013). Weight loss equal or above 10% of body weight was not associated with other symptoms, smoking habits, alcohol consumption, extent of the disease, location and other endoscopic appearance of the lesions (p>0.05).

The analyses of association between the variables dyspnea on exertion and the topographical features are displayed in Table 5. There was no significant difference in the occurrence of dyspnea at rest between the groups of individuals with uni- or bilateral lesions, with vocal fold paresis or paralysis and between patients grouped by the number of affected sites (p>0.05). No association was observed between presence of dyspnea at rest and presence of dyspnea on exertion with smoking habits (p>0.05).

**Table 5:** Comparison of the presence or not of dyspnea on exertion with topographical features of the lesions of 36 patients with laryngeal tuberculosis. Evandro Chagas National Institute of infectious diseases—FIOCRUZ, 2004 to 2014.

	Presence of dyspnea on exertion				
	Yes		No		
	(N = 19)		(N = 17)		
Laryngeal topographical features	n	%	n	%	p-valor
Lesions in the epiglottis	13	68.4	8	47.1	0.194^b^
Lesions in the aryepiglottic fold	15	78.9	7	41.2	**0.020**^b^
Lesions in the arytenoid region	12	63.2	6	35.3	0.095^b^
Lesions in the interarytenoid region	8	42.1	4	23.5	0.238^b^
Lesions in the false vocal fold	16.7	84.2	8	47.1	**0.018**^b^
Lesions in the true vocal fold	18	94.7	14	82.4	0.326^a^
Lesions in the subglottic region	3	15.8	1	5.9	0.605^a^
Lesions in four or more sites	15	78.9	10	58.8	**0.020**^b^
Bilateral lesions	18	94.7	10	58.8	**0.016**^a^

N—total number, n—valid number, **bold—significant p value**

^a^—p-value calculated by the Fisher`s Exact Test

^b^—p-value calculated by the Pearson’s Chi-Square Test

The association between smoking habits and coughing could not be evaluated because all smokers had this symptom. All smokers presented bilateral lesions. In addition, lesions in four or more sites were more frequent among smokers (81.2%) than among non-smokers (45%) (p = 0.041). Tobacco use was not associated with the endoscopic appearance of the lesions (p>0.05). Also, we did not find association between location, extent and endoscopic appearance of laryngeal lesions with alcoholism or concomitant use of tobacco and alcohol.

## Discussion

In this study, when assessing one of the largest series of cases described of LTB, we observed that dysphonia and odynophagia are associated with the location of LTB lesions, that odynophagia as first symptom and ulcerated lesion are associated with weight loss and, that smoking, dyspnea on exertion, cough and odynophagia are associated with a greater extension of the laryngeal lesions.

The predominance of men in the fifth or sixth decade of life, with no significant age difference between genders, observed in our study, has been frequently reported [16,20,21], although a lower age of female patients has been described [16]. Most of the patients had low socioeconomic conditions, such as low income and low education level which were factors described as facilitators for the development of the disease [15,22,23].

As noted in our study, LTB patients may present history of previous pulmonary TB treatment [10,16,24] and, although a long evolution of the disease, with periods of up to two years, has already been described [7,16,25], the observation that this time is higher in patients with dysphonia is unprecedented. Probably this symptom is undervalued by sick people who take longer to seek for medical attention. On the other hand, we observed that symptoms such as odynophagia occur with greater discomfort, decreased food intake and consequent weight loss which can reduce the time for seeking for medical help [26,27]. In the same manner, the lack of specific symptoms [3] and the insidious nature of LTB, together with the difficult access of patients to medical attention and specialized exams [14], may delay diagnosis. However, similarly to Wang *et al* (2007), we found no evidence that the delay in the diagnosis predisposes the patient to larger laryngeal lesions [16]. It is possible that the extent of the laryngeal lesion is not necessarily part of the natural history of the disease, but a more serious outcome in some patients [26].

The true vocal folds are directly related to voice quality, once they are the oscillatory component of voice production [28]. As the true vocal folds are the main affected site in TBL, dysphonia is usually observed as the main symptom [13,16,20,29]. In a previous study our group described the voice quality in LTB, showing that voice disorders found in active LTB are similar to that reported after clinical healing of the disease [29]. In the present study we extended our analysis to the clinical, epidemiological and topographical features aiming to better characterize the LTB in order to improve information that would help during the LTB diagnosis.

The higher frequency of cough found in our study may be related to a greater concomitance of LTB with pulmonary TB, different from others, where the frequency of this symptom was smaller, probably because they had a higher percent of isolated LTB [20,25,30,31]. The association between cough and bilateral lesions shows that this symptom is related to a more extensive larynx involvement, probably triggering afferent stimuli in a greater number of cough receptors of this organ. In accordance with this hypothesis, we observed that all patients with lesions in four or more laryngeal sites had cough. In addition, all the smokers that participated in our study had cough. As TB patients are often smokers [24,25,32], and cough is common in smokers, this symptom does not usually draw the attention of these patients delaying their search for medical assistance, leading to delays in diagnosis confirmation of LTB [11].

We also observed a higher frequency of odynophagia than usually reported [13,16,20], besides it being associated with the involvement of the epiglottis, aryepiglottic folds and arytenoid regions. These laryngeal sites have greater contact with the food bolus and their movement during swallowing leads to a painful condition. It is assumed that the intensity of odynophagia is related to the degree of laryngeal lesions, mainly of the epiglottis [33]. In addition, we observed association of this symptom with higher number of laryngeal sites, and also its presence in all patients with lesion extended to the pharynx, suggesting that more extensive lesions have greater potential for generation of painful stimuli.

It can be suggested that the associations found between odynophagia, ulcerated lesion, epiglottis lesion and weight loss are part of a sequence of events with causal link, in which ulcerated lesions in the epiglottis cause odynophagia, which in turn, reduces food intake thus causing weight loss. This association between painful lesions in upper aerodigestive tract with weight loss has already been reported in cases of tegumentary leishmaniasis [27].

Dyspnea on exertion was associated with extent of the laryngeal lesions, once this symptom was more frequently found among patients with bilateral involvement or involvement of four or more anatomic sites of this organ, probably due to greater narrowing of larynx lumen, reducing the breathing space. On the other hand, dyspnea at rest seems to relate better with the degree of lung involvement of TB, because we did not detect association between this symptom and the extent of the laryngeal lesion.

Tobacco use has been widely accepted as TB determinant factor, because it alters all the defense mechanisms of the respiratory tree and reduces oxygen concentration in blood, enhancing lesion severity [1,34]. However, the association between smoking habits and LTB lesions in four or more sites had not yet been described. Considering this association and the fact that, in our study all the smokers had bilateral laryngeal involvement, we suggest that smoking be considered a risk factor for the development of more extensive lesions, since, as described in this paper, the extent of LTB lesions is not directly associated with the duration of disease progression.

In our study, the most frequent endoscopic presentation was a lesion of granulomatous appearance. Due to the lack of standardization of the endoscopic findings, it is difficult to compare our results with those in the literature. In the 1980s, a classical description consisted in multiple lesions, frequently ulcerated [21]. Currently, ulcerated lesions are still observed [13,25,35], but some authors suggest that the lesions are hypertrophic or exophytic in most cases, and difficult to distinguish from chronic laryngitis of other etiologies [10,15,35]. Considering that the endoscopic appearance of the lesions is nonspecific and varied, and can mimic other laryngeal diseases, LTB diagnosis requires high degree of suspicion [24,25,33].

According to our results and other reported in the literature, the true vocal folds are usually the most frequently affected anatomic site [16,20,21,25], although some studies do not report preference for any portion of the larynx [11,13]. Our study, like others that identified higher incidences of epiglottis involvement, reports a percentage of concomitant pulmonary TB higher than 80% [20,36]. Based on this observation and the fact that the glosso-epiglottic valleculae, adjacent to the epiglottis, present higher potential for residue accumulation, we suggest the hypothesis that secretions from the lower airways can remain in higher amount and for longer periods of time restrained there, contributing to the development of infection in this laryngeal structure. Similarly, the highest percentages of concomitant involvement of more than one larynx anatomic structure and bilateral involvement of this organ in the present study can be related to the prevalence of pulmonary TB, as previously suggested [10,20]. The classic involvement of the posterior portion of the larynx due to accumulation of infected secretion in bedridden patients, although still observed [7,15,31], is no longer a cardinal sign of LTB as it was in the past [12].

It is believed that LTB originates in the hematogenous spread from a distant primary focus (hematogenous theory) or by direct spread of bacilli in bronchial secretions (bronchogenic theory) [12,16,21]. Our observation of a pulmonary infection focus in 97.2% of LTB patients supports that direct spread from a bronchial focus is the most frequent mechanism, in agreement with most of the reviewed studies [16,20,21]. Thus, chest X-ray is an important complementary examination, since images suggestive of pulmonary TB in individuals with laryngeal lesions raise the suspicion of LTB [12,16].

The skin test with PPD presented variable results in different studies (45% to 90.9%) [13,24,25,30,37], and a negative result does not exclude the presence of LTB [15,30,35]. In addition, although with no association, the fact that all the patients in this study with negative PPD had involvement of four or more laryngeal sites, makes us suppose that the more extensive the infection, the more likely the compromise of the immune response leading to a negative result in the test. In the same way, we observed no association between bacterial load and PPD.

The positivity of sputum direct examination in this study was similar to that observed in the literature [20,32,36], but the higher frequency of positive results in sputum culture in our study [13,16,20] may be related to the fact of having a higher number of patients with associated pulmonary TB, beyond the fact that INI-FIOCRUZ is a reference center for the diagnosis of tuberculosis and other infectious and parasitic diseases, which can contribute to a higher percent of positive sputum due to the expertise of the local laboratory and the professionals that help the patient to collect a suitable specimen. However, despite this higher positivity, we did not find association between bacterial load and greater LTB extent. The positivity of both, bacilloscopy and culture of laryngeal tissue fragment well below that of sputum suggests that the lung focus is the direct responsible for the elimination of mycobacteria through the sputum. The fact that the single patient with no lung involvement presented negative bacilloscopy by the Ziehl-Neelsen technique and culture in both, sputum and laryngeal tissue fragment, corroborates our hypothesis. Still in this case, although BAAR were identified by Wade stain in the fragments, they were described as rare. On the other hand, the negative results of the bacteriological tests in this LTB case without concomitant lung focus demonstrates the importance of the laryngeal biopsy to obtain tissue specimens, whose histopathologic studies, together with clinical features, can confirm the diagnosis of the disease.

In the histopathologic examination of the specimens obtained by laryngeal biopsy we observed percentages of granulomatous, chronic inflammatory processes similar to those reported in the literature [7,25,36]. In non-immunosuppressed patients tissue bacilloscopy is usually negative and the presence of granuloma with caseous necrosis suggests TB [1].

We also observed some cases of impaired laryngeal mobility. Vocal fold paralysis is an aspect that LTB shares with laryngeal cancer, and its presence raises the suspicion of malignant neoplasm, impairing the differential diagnosis [12,16,31]. In addition, the coexistence of malignant neoplasm of the larynx and pulmonary TB has been described [21,30], enhancing the importance of LTB diagnosis through histologic examination.

Even though the WHO suggests that LTB may be included with pulmonary TB for the purpose of reporting, given the high degree of infectiousness associated with many of LTB cases [38], we are convinced that many cases of LTB have not been diagnosed either by the facility to confirm TB by pulmonary lesions or the by the difficulties to confirm the laryngeal involvement. Corroborating this classification into pulmonary TB, the Brazilian Ministry of Health recommends using the basic scheme with TB drugs for 6 months for treatment of cases of LTB [1], despite being reported in the literature that the treatment time should be frequently longer than 6 months and may be extended to 1 year [7,16,31,32,37]. Especially in cases of LTB without concomitant pulmonary involvement, this diagnostic hypothesis is not always raised by physicians. Therefore, the purpose of a sub-classification of TB into LTB will allow the patient to be subjected to specialized follow up and appropriate treatment, since the laryngeal focus has to be monitored by videolaryngoscopy and requires drug therapy usually for a longer period than the one used for pulmonary TB. In this connection, the best characterization of clinical, endoscopic and laboratory features of LTB can allow the identification of the laryngeal involvement by TB. The case of LTB without concomitant pulmonary TB lesion detected in our study confirms this possibility. The knowledge concerning the clinical features and the most common endoscopic characteristics of LTB and its associated factors identified in this study may guide physicians to continue the diagnostic investigation for LTB, through laryngeal biopsies and histopathological studies, even with negative microbiological tests for TB. This study improves the understanding of LTB and could help physicians raise the hypothesis of TB in the presence of chronic laryngeal lesions and consequently facilitates its differential diagnosis. Even though the lesions and symptoms described in the present study cannot be considered as specific features of LTB, our results point to the necessity of differential diagnosis since we demonstrate that they are also present in LTB. As the hypothesis of LTB diagnosis is rarely considered by otorhinolaryngologists, our results can draw attention of physicians to the need for TB inclusion in the differential diagnosis of laryngeal lesions. The absence of this inclusion may delay its diagnosis and consequently increase the incidence of complications.

We did not find studies in the literature describing the factors associated with clinical and topographical features of LTB as reported in the present study. Our results suggest that smoking is associated with the development of more extensive LTB lesions. In turn, the extent of the laryngeal lesions is also associated with dyspnea on exertion and to odynophagia with consequent impairment of the nutritional status. Thus, smoking seems to be an important factor in the development of more severe manifestations of LTB. On the other hand, in relation to the diagnosis of this disease, it is important to emphasize the need for histopathologic confirmation, because the positivity of sputum bacteriologic examination seems not to necessarily reflect laryngeal involvement.
